# Nursing strategies for implementing psychosocial interventions to address violence behavior in schizophrenia: a scoping review

**DOI:** 10.1186/s12912-025-03145-2

**Published:** 2025-05-08

**Authors:** Iyus Yosep, Rohman Hikmat, Suryani Suryani, Efri Widianti, Aat Sriati, Titin Sutini, Imas Rafiyah

**Affiliations:** 1 Department of Mental Health, Faculty of Nursing, Universitas Padjadjaran, Sumedang, Jawa Barat Indonesia; 2 Nursing Department, Faculty of Health Science, Universitas ‘Aisyiyah Bandung, Bandung, Jawa Barat Indonesia

**Keywords:** Interventions, Patients, Schizophrenia, Violent behavior

## Abstract

**Background:**

Psychosocial interventions are crucial in managing violent behavior problems in people with schizophrenia, considering the high risk to self and others. Although drug therapy plays an important role, psychotherapy approaches offer holistic solutions in reducing violent behavior that is complex and often resistant to treatment. Therefore, a comprehensive review of the literature on these psychosocial interventions is necessary to evaluate the various approaches that have been developed.

**Objective:**

This study aims to map and synthesize existing literature on psychosocial interventions designed to reduce violent behavior in patients with schizophrenia.

**Methods:**

A scoping review was carried out by searching for articles from the CINAHL, PubMed, and Scopus databases using the keywords “schizophrenia”, “nursing”. “psychosocial intervention”, and “violence”. Inclusion criteria included studies published in English using original research, reporting the results of a nursing intervention, full text, and a publication period of the last five years (2019–2024). Data was extracted using manual tables, and analysis was carried out descriptively qualitatively.

**Results:**

There were 12 articles that met the inclusion criteria and discussed various psychosocial interventions to reduce violent behavior in people with schizophrenia. The results showed that five types of nursing strategies, logotherapy, assertive therapy, forgiveness therapy, cognitive behavioral therapy (CBT) and social skills training (SST), and assertive communication and de-escalation training, consistently resulted in significant reductions in violent behavior.

**Conclusion:**

This scoping review underscores the need for a comprehensive approach to managing violent behavior in schizophrenia by utilizing psychosocial interventions that have been proven to be effective. However, the limited number of studies, heterogeneity in intervention methods, and variability in outcome assessments warrant careful interpretation of the results. Nursing implications include improvements in training and support for nurses to implement these interventions in daily clinical practice. Recommendations for future research include the need for more in-depth studies to explore effective intervention mechanisms as well as improvements in methodologically more robust study designs.

## Introduction

Schizophrenia is a serious mental disorder that affects approximately 1% of the world’s population [1]. Schizophrenia is known for symptoms such as disturbed thinking, distorted perception, and difficulty in distinguishing between reality and hallucinations [2]. While most individuals with schizophrenia are not violent, a small subset of patients, particularly those with comorbid substance use or inadequate treatment, may exhibit aggressive behavior. This behavior, though relatively uncommon, can present challenges in clinical management and may impact the well-being of both patients and their surrounding environment [3]. The prevalence of schizophrenia in the United States is estimated to be approximately 0.25–0.64% of the population [3]. In Europe, the prevalence of schizophrenia ranges from 0.4 to 0.6%, with significant differences between countries such as Sweden, which reports lower rates, and Ireland, which reports higher rates [4]. In Asia, schizophrenia prevalence rates also vary, with estimates of around 13.8% [5].

Violent behavior in schizophrenic patients is a serious, complex problem, influenced by factors such as uncontrolled psychotic symptoms, substance or alcohol use, and past traumatic experiences [6]. Some of the risk factors that have been identified include the presence of uncontrolled psychotic symptoms such as hallucinations or delusions that cause fear or a sense of threat, a history of substance or alcohol use that can worsen symptoms, and past experiences of undiagnosed or untreated trauma [7]. The impact of this violent behavior includes a high risk of physical injury and psychological trauma to the patient, while families often experience emotional stress and difficulty providing adequate care [8]. At the societal level, violent behavior can increase safety concerns, exacerbate the stigma of mental disorders, and reduce overall quality of life [9].

Psychosocial intervention is a therapeutic approach that includes various strategies to improve the psychological and social well-being of individuals, with the main aim of increasing their adaptation to the surrounding environment. In people with schizophrenia, psychosocial interventions include approaches such as individual or group counseling, social skills training, family support, and psychosocial rehabilitation aimed at improving the patient’s social, occupational and independent functioning [10]. This approach not only aims to reduce psychotic symptoms, but also to improve the patient’s quality of life and facilitate their optimal reintegration into society [11].

Nurses play a crucial role in implementing nursing strategies to manage violent behavior in patients with schizophrenia. These strategies include conducting comprehensive risk assessments, identifying triggering factors, and designing appropriate prevention measures [12, 13]. Nurses also employ therapeutic communication as part of their nursing strategies to build trusting relationships with patients, enhance self-awareness, and teach adaptive coping skills. Specific nursing strategies such as social skills training, forgiveness therapy, and assertive communication are utilized to support patients in developing prosocial behavior [14]. In addition, nurses implement educational strategies for families to promote effective patient support and actively collaborate with multidisciplinary teams to ensure a comprehensive and sustainable approach in preventing violent behavior [15].

Previous studies have demonstrated the effectiveness of these nursing strategies in reducing violent behavior among patients with schizophrenia, emphasizing the pivotal role of nurses as frontline providers [16, 17]. For instance, structured social skills training programs facilitated by nurses have significantly reduced violent tendencies while enhancing patients’ social interaction abilities [18]. Similarly, nurse-led family-based strategies have proven effective in reducing the frequency and intensity of violent behavior by strengthening family involvement [19]. Community-based nursing strategies, including nurse-led outreach and engagement programs, have also contributed to reduced violence by promoting social connectedness and decreasing isolation. Moreover, the integration of cognitive-behavioral approaches into nursing care plans, particularly through psychoeducation and stress management techniques, has been beneficial in helping patients control psychotic symptoms and manage triggers of violent behavior [20].

A systematic review has further highlighted the role of psychosocial interventions in reducing both positive and negative symptoms of schizophrenia, including the risk of violent behavior [21]. This underscores the need for research focusing specifically on psychosocial interventions that target violent tendencies within the context of nursing strategies. Addressing this issue is essential not only for improving patients’ quality of life and minimizing the social consequences of violent behavior but also for equipping nurses with evidence-based strategies to enhance clinical and community practices. Therefore, this scoping review aims to map and synthesize existing evidence on nursing strategies for psychosocial interventions designed to reduce violent behavior in patients with schizophrenia.

## Materials and methods

### Study design

This study used a scoping review approach in accordance with the framework developed by Arksey & O’Malley [22]. This approach was chosen because it allows for a comprehensive exploration of the existing literature on psychosocial interventions to reduce the risk of violent behavior in people with schizophrenia, as well as allowing for the inclusion of studies with diverse designs. The stages of this scoping review approach include: (1) Formulating research questions, (2) Identifying relevant studies, (3) Selecting appropriate studies, (4) Extracting relevant data, and (5) Reporting or synthesizing the results. The research question are what types of psychosocial interventions are effective in reducing violent behavior among people with schizophrenia, and what are their implications for nursing practice?

### Search strategy and eligibility criteria

A literature search was conducted using the Scopus, PubMed, and CINAHL databases with keywords combining Boolean operators and MeSH terms where applicable. Scopus, PubMed, and CINAHL were selected because they provide broad coverage of high-quality, peer-reviewed literature in nursing, psychiatry, and healthcare. PubMed was chosen for its extensive biomedical and clinical research content, CINAHL for its focus on nursing and allied health sciences, and Scopus for its multidisciplinary coverage, which captures a diverse range of psychosocial intervention studies.

The main research question was to identify what psychosocial interventions have been studied to reduce the risk of violent behavior in people with schizophrenia, with a particular focus on their implications for nursing practice. Primary keywords included “schizophrenia,” “psychosocial intervention,” “violence risk,” and “nursing” or “nurses.” The search strategy was tailored for each database to enhance sensitivity and specificity. In PubMed and CINAHL, appropriate Medical Subject Headings (MeSH and MH terms) were used to improve search precision.

The search strings used were:

Scopus: (TITLE-ABS-KEY(“schizophrenia” OR “psychotic disorders” OR “psychosis”) AND TITLE-ABS-KEY(“psychosocial intervention” OR “psychosocial support” OR “psychological therapy”) AND TITLE-ABS-KEY(“violence risk” OR “aggression” OR “violent behavior” OR “risk of violence”) AND (TITLE-ABS-KEY(“nursing” OR “nurses” OR “nursing strategy” OR “nursing role”)))

PubMed: ((“Schizophrenia“[MeSH] OR “schizophrenia“[All Fields] OR “psychotic disorders“[MeSH] OR “psychosis“[All Fields]) AND (“Psychosocial Intervention“[All Fields] OR “Psychosocial Support systems“[MeSH] OR “Psychological Therapy“[All Fields]) AND (“Violence Risk“[All Fields] OR “Aggression“[MeSH] OR “Violent Behavior“[All Fields] OR “Risk of Violence“[All Fields]) AND (“Nursing“[MeSH] OR “Nurses“[All Fields] OR “Nursing strategy“[All Fields] OR “Nursing role“[All Fields]))

**Fig. 1:**
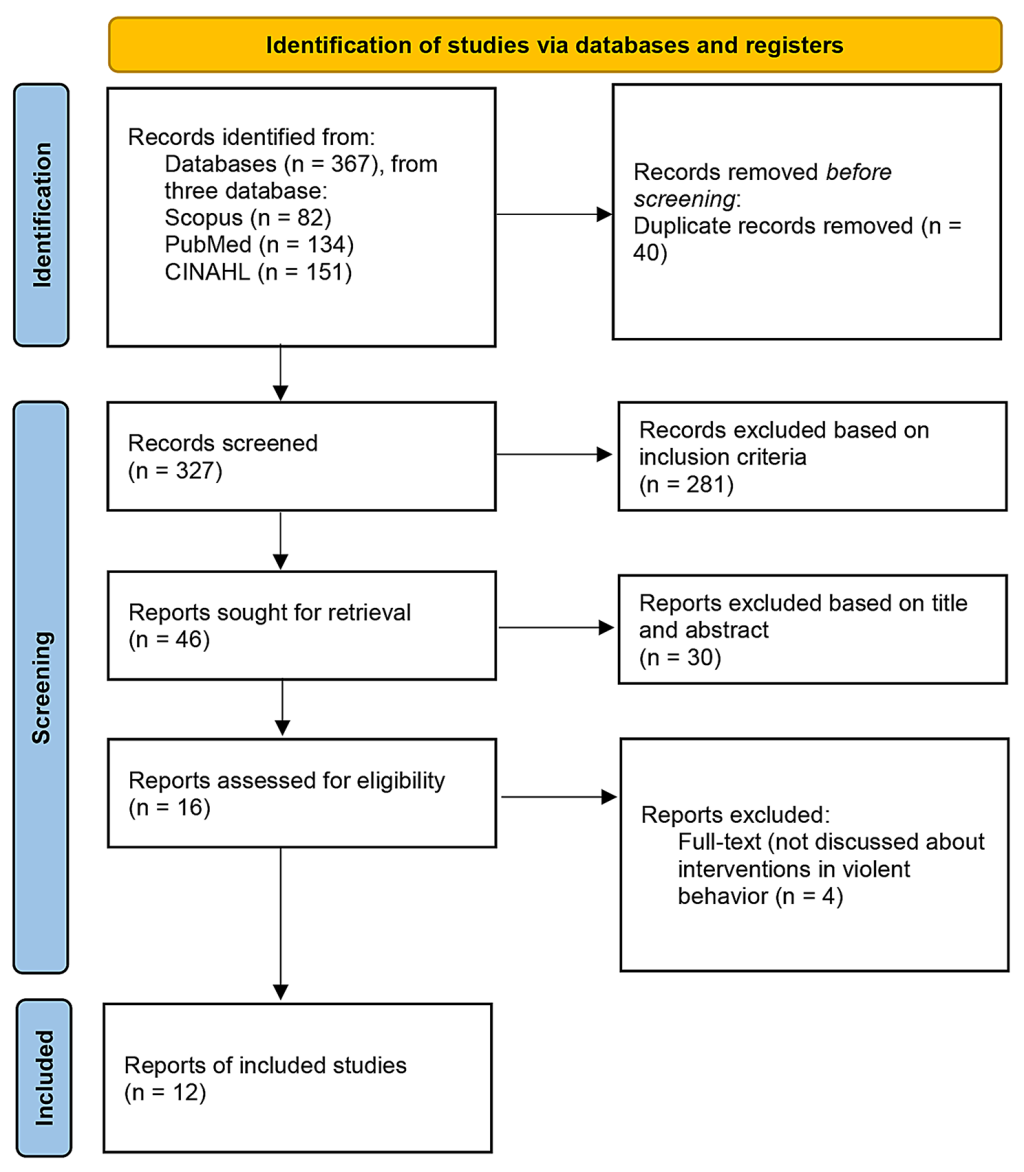
PRISMA flow diagram

CINAHL: ((MH “Schizophrenia” OR “schizophrenia” OR “psychotic disorders” OR “psychosis”) AND ((MH “Psychosocial Intervention”) OR “psychosocial intervention” OR “psychosocial support system” OR “psychological therapy”) AND ((MH “Violence Risk”) OR “violence risk” OR “aggression” OR “violent behavior” OR “risk of violence”) AND ((MH “Nursing”) OR “nursing” OR “nurses” OR “nursing strategy” OR “nursing role”)) (Fig 1).

### Inclusion and exclusion criteria

Inclusion and exclusion criteria in this scoping review were developed using the PCC (Population, Concept, Context) framework to ensure alignment with the research objectives. The population of interest was individuals diagnosed with schizophrenia. The concept focused specifically on psychosocial interventions aimed at reducing the risk of violent behavior, rather than general therapeutic approaches. The context encompassed studies conducted in healthcare or community-based settings across various countries. Additional inclusion parameters included original research articles published in English, with full-text availability, and a publication year between 2019 and 2024. Only studies that discussed or evaluated psychosocial interventions with implications for nursing practice were included. This focus ensured that the scope remained centered on psychosocial strategies relevant to nursing without restricting the search to studies labeled exclusively as “nursing interventions,” thus maintaining a broader yet still applicable range of literature.

Studies were excluded if they did not involve psychosocial interventions, did not target individuals with schizophrenia, or failed to discuss outcomes related to violent behavior. Articles with vague or non-specific descriptions of the intervention, those that lacked any reference to nursing roles or implications, and publications not written in English were also excluded. Furthermore, review articles, editorials, opinion pieces, and commentaries were not considered. To reduce excessive “noise” in the search results, studies that used overgeneralized or non-specific terms such as “therapy” or “care” without clearly defining their psychosocial nature or relevance to the schizophrenia population were also excluded.

To ensure systematic data management, a reference manager software Mendeley dekstop was used to organize and track eligible studies throughout the screening process.

### Data extraction

Data extraction was conducted systematically using a structured table created manually in Microsoft Excel. The table included the following elements: authors, study aims, research design, sample characteristics, country, tools or questionnaires used (if applicable), psychosocial interventions implemented, the role of nurses, and the results or outcomes reported. The extraction process was performed independently by two researchers who were trained and experienced in nursing and psychosocial research. In cases of differing opinions, discussions were held to reach a consensus. If no agreement was achieved, a third researcher was consulted to make a final decision. This rigorous approach ensured consistency and accuracy in capturing data related to psychosocial interventions and their implications for nursing practice.

### Data analysis

Data analysis was carried out descriptive qualitatively with a thematic analysis approach to identify and describe main themes based on research results. The analysis stages include (1) Selection of relevant studies, (2) Data coding to identify themes, (3) Development of theme categorization, (4) Interpretation and presentation of results in the context of psychosocial interventions on the risk of schizophrenic violent behavior.

To enhance the rigor of the study, data analysis was conducted independently by two authors with expertise in nursing and psychosocial interventions, ensuring reliability in theme identification. Investigator triangulation was applied through the involvement of a third researcher to review and validate themes in cases of significant discrepancies. An audit trail was maintained to document the decision-making process, coding framework, and theme development, ensuring transparency and replicability. Regular discussions among the research team were also conducted to minimize subjective bias and enhance consistency in theme interpretation. These measures strengthened the credibility, dependability, and confirmability of the thematic analysis in this scoping review.

## Results

Based on the results of the initial search, the authors obtained 367 reports. After removing 40 duplicate articles, 327 articles remained. The authors then applied the inclusion criteria and found that 281 articles did not meet the eligibility criteria. After screening titles and abstracts, 30 more articles were excluded as they did not align with the research topic. A full-text review of 16 articles was conducted, and four articles were excluded due to a lack of discussion on psychotherapy interventions to reduce the risk of violent behavior in people with schizophrenia. As a result, 12 articles were included in the final analysis.

These findings emphasize that psychosocial interventions, when implemented by competent and trained nurses, can serve as powerful nursing strategies to reduce violent behavior and promote psychological well-being in patients with schizophrenia. Integrating these interventions into nursing strategies requires not only knowledge of therapeutic techniques but also strong communication skills, emotional sensitivity, and clinical judgment. Nurse educators and healthcare institutions should prioritize training and supervision to ensure the delivery of effective psychosocial interventions in both inpatient and community mental health settings. There was significant heterogeneity in the intervention types, ranging from logotherapy, assertive therapy, forgiveness therapy, cognitive behavioral therapy (CBT) and social skills training (SST), to assertive communication and de-escalation training, making it difficult to generalize findings across different healthcare (Table 1).

**Table 1:** Extraction data

No	Author, Year	Objective	Country	Design	Sample	Intervention	Results
1.	(mahmoudfakhe, 2022)	Investigating the effectiveness of group logotherapy on psychological distress and belief in a just world in patients	Iran	Quasi experiment	30 patients	Group logotherapy consists of eight sessions. These sessions include group introduction, therapeutic awareness of meaning, understanding the meaning of life, individual freedom and responsibility, the meaning of love and personal experience, the meaning of suffering, the meaning of death, and summarizing the findings.	The study found significant differences in psychological distress, and belief in a just world between the experimental and control groups.
2.	(Saswati, 2020)	Examining the effects of logotherapy in increasing self-awareness and meaning in life and reducing violent behavior in schizophrenic clients.	Indonesia	Quasi experiment	74 respondents	Logotherapy consists of 4 sessions, each with 2 meetings lasting 60 min. These sessions include problem identification, stimulation of creative imagination, meaningful situations, and meaning in life.	Logotherapy significantly improved self-esteem, violent behavior, and meaning in life in the intervention group.
3.	(Siregar et al., 2020)	Comparing the differences between Cognitive Behavioral Therapy (CBT) and Assertive Training (AT) in controlling violent behavior in schizophrenia patients.	Indonesia	quasi experiment	30 respondents	Assertive therapy includes discussion of violent behavior, assertive behavior training, use of group support systems, and support evaluation. This therapy improves assertive communication skills and motivates clients to be more active.	CBT is more effective than AT in controlling patients’ violent behavior.
4.	(Rismarini & Hasanat, 2022)	Investigating the effect of Forgiveness Therapy on the psychological well-being and violent behavior of schizophrenic patients.	Indonesia	Case studies	3 participants	Forgiveness Therapy uses Koeswardhani’s (2011) manual and includes six 120-minute sessions, twice a week. Topics include uncovering negative emotions, facing deepest emotions, deciding to forgive, working on forgiveness, developing positive emotions, and setting new life goals.	Forgiveness Therapy improves the psychological well-being and violent behavior of schizophrenia patients.
5.	(Praptomojati & Subandi, 2020)	Investigating the effect of Forgiveness Therapy on increasing self-acceptance and violent behavior in patients	Indonesia	quasi-experimental	seven adult inmates	CBT intervention is carried out by identifying unpleasant experiences and changing them into positive thoughts and behavior. SST sessions include techniques for getting acquainted, making friendships, working in groups, dealing with difficult situations, and evaluating the benefits of therapy.	Forgiveness Therapy increases self-acceptance and violent behavior
6.	(Suhron et al., 2020)	Analyzing the effects of emotion-focused forgiveness therapy on violent behavior in post-incarceration schizophrenia.	Indonesia	Quasi experiment	64 patients with violent behavior	Assertiveness training helps participants recognize and overcome passive/aggressive behavior, as well as practice assertive statements and nonverbal techniques for effective communication. Evaluation includes direct feedback and reflection on participants’ learning experiences.	Remission therapy is more effective than emotional therapy in reducing schizophrenic violent behavior.
7.	(Fitriani et al., 2021)	Investigating the effects of cognitive behavioral therapy (CBT) and social skills training (SST) on signs and symptoms of risk for violent behavior.	Indonesia	Quasi experiment	30 patients with symptoms of risks of violent behavior	Three treatment groups were given AT, ACT, and AACT interventions, while the control group followed hospital standards. Evaluation was carried out by observation using a violent behavior measurement scale for 3 days.	CBT and SST significantly reduce signs and symptoms of violent behavior in patients.
8.	(Fahrizal et al., 2020)	Presents changes in signs, symptoms and the patient’s ability to manage the risk of violent behavior after being given assertive training therapy and family psychoeducation using Roy’s theoretical approach.	Indonesia	Case reports	11 patients	Assertive communication training involves stages of description, learning, practice, and role playing. This research optimizes the role-playing stage to control anger and prevent recurrence of violent behavior.	Assertiveness training and family psychoeducation reduce violent behavior and increase the ability to overcome risks.
9.	(Rustafariningsih et al., 2019)	Analyzing the effect of AACT on violent behavior in schizophrenia patients.	Indonesia	Quasi experiment	32 respondents	The supportive assertive group therapy program is carried out in groups of 2–3 patients for 4 sessions, each session lasting 45–60 min. After therapy, subjects were given food containing anthocyanins for 10 days.	AACT is more effective than AT and ACT in reducing patient violent behavior.
10.	(Endang Nihayati et al., 2020)	Analyzing the effect of role playing on the ability to control anger in schizophrenia with violent behavior in society.	Indonesia	Quasi experiment	36 participants	The de-escalation training has a total duration of 16 h consisting of theory and workshop sections. Material includes de-escalation techniques, verbal and non-verbal communication, and handling emotional responses.	Assertiveness training improves anger control abilities in schizophrenic patients with violent behavior.
11.	(Avianti et al., 2020)	Analyzing the effects of anthocyanins and supportive assertive group therapy on the ability to overcome violent behavior in schizophrenia patients at the West Java Provincial Hospital.	Indonesia	Quasi experiment	36 patients	Group logotherapy consists of eight sessions. These sessions include group introduction, therapeutic awareness of meaning, understanding the meaning of life, individual freedom and responsibility, the meaning of love and personal experience, the meaning of suffering, the meaning of death, and summarizing the findings.	Supportive assertive group therapy is effective in dealing with violent behavior in schizophrenia patients.
12.	(Celofiga et al., 2022)	Assessing the effects of verbal and non-verbal de-escalation on the incidence and severity of aggression and use of physical restraint in acute psychiatric wards.	Slovenia	RCT	3,211 participants	Logotherapy consists of 4 sessions, each with 2 meetings lasting 60 min. These sessions include problem identification, stimulation of creative imagination, meaningful situations, and meaning in life.	De-escalation training effectively reduces aggression and use of physical restraint in an acute psychiatric unit.

### Logotherapy

Group logotherapy, which consists of eight sessions, shows a significant reduction in psychological stress and an increase in belief in a just world in the experimental group compared to the control group [23]. Logotherapy with four sessions, each lasting 60 min, has been shown to significantly improve self-esteem, meaning in life, and reduce violent behavior in people with schizophrenia. Nurses can support patients in exploring existential issues, identifying personal values, and reconstructing life meaning, which may reduce aggression and foster psychological adaptation [24, 25]. This approach can reduce frustration and aggression, which often emerge in people with schizophrenia. The results of this therapy show a significant increase in the understanding of the meaning of life and better psychological adaptation for participants, providing deep insight into human existence and its relevance in facing life’s challenges [26].

### Assertive therapy

Assertive therapy, which includes discussions about violent behavior, assertive communication exercises, and group support evaluation, has proven effective in enhancing patients’ assertive communication skills and motivating them to be more active in the therapy process [27]. This intervention shows significant effectiveness in reducing violent behavior in people with schizophrenia, and its relevance in nursing practice is considerable. This intervention consists of a series of specially designed sessions, starting with a description of the new behavior that needs to be learned, learning through demonstration, direct exercises in the group, as well as role-playing to practice possible scenarios. The goal of each session is to help people with schizophrenia improve assertive communication skills, identify anger triggers, and respond to them in a more constructive and controlled way [3]. For nurses, this strategy involves guiding patients through structured sessions using role-playing and scenario-based learning to develop assertive communication. Trained psychiatric nurses are in a unique position to deliver assertive therapy as part of therapeutic group programs, helping patients recognize anger triggers and apply coping strategies in a controlled manner [8].

### Forgiveness therapy

Forgiveness therapy consists of six sessions, which include the exploration of negative emotions, the forgiveness process, and the development of positive emotions. This intervention is structured in several sessions that involve deep emotional processes, such as expressing negative emotions related to their experiences, deciding to forgive, and developing new perspectives on the conditions faced [28]. Each session is designed to achieve a specific goal, namely helping caregivers manage stress and improve their quality of life through better self-understanding and acceptance. The research results indicate that this intervention can improve patients’ psychological well-being and significantly reduce violent behavior. As a nursing strategy, competent psychiatric nurses can utilize this intervention by facilitating discussions around unresolved emotional pain and guiding patients toward forgiveness and reconciliation. This approach fosters emotional regulation and can significantly lower hostility.

### Cognitive Behavioral Therapy (CBT) and Social Skills Training (SST)

Cognitive Behavioral Therapy (CBT) for the management of violent behavior in patients with schizophrenia involves a series of structured sessions [27]. This intervention consists of several sessions with an approach that focuses on identifying automatic negative thoughts, changing thought patterns through CBT techniques, providing support from systems within and outside the family, and evaluating the benefits of the therapy applied [29]. In addition, Social Skills Training (SST) includes social interaction techniques, such as building friendships and handling difficult situations, which have also proven effective in controlling violent behavior [30]. In nursing practice, these interventions can be embedded within psychosocial rehabilitation programs. Competent nurses trained in CBT and SST techniques can help patients navigate social interactions, interpret situations more accurately, and respond with appropriate behavior, thus minimizing the risk of violent outbursts.

### Assertive communication and de-escalation training

Assertive communication and de-escalation training using description methods, role-playing exercises, and reflection on experiences helps control anger and prevent the recurrence of violent behavior. This 16-hour training program has been proven effective in reducing aggression and the use of physical restraints in acute psychiatric units. Assertive communication and de-escalation training are highly relevant for nurses working with people with schizophrenia, particularly in managing aggression or anger in psychiatric units. This intervention consists of a series of sessions that include a theoretical review of aggressive behavior, a workshop that teaches de-escalation techniques using video materials and role plays, and the use of a manual as a learning guide [31]. The main aim of this training is to provide psychiatric staff with an in-depth understanding of the techniques that can be used to de-escalate situations of aggression, both verbal and non-verbal, so as to prevent escalation of violence. Nurses play a pivotal role in both delivering and modeling assertive communication strategies. Trained nurses can lead de-escalation training workshops, supervise patients during emotionally charged moments, and promote a therapeutic environment conducive to nonviolent conflict resolution.

### Scientific gap

Although various types of psychosocial therapies, such as logotherapy, cognitive behavioral therapy (CBT), assertive training, and forgiveness therapy, have proven effective, there is a tendency for research to focus on one type of intervention without comparing or combining different approaches that could complement each other. Most studies use quasi-experimental research designs with relatively small samples and are limited to specific locations, so the results may not be generalizable to a larger population. Moreover, many studies do not provide sufficient insight into how nurses can effectively integrate these interventions into daily nursing practice in various settings, such as hospitals, clinics, or communities. Although some studies show the short-term success of psychosocial interventions, there is almost no research exploring the long-term impact of these interventions on violent behavior in people with schizophrenia.

## Discussion

The results of this scoping review indicate that psychotherapy interventions show potential in reducing the risk of violent behavior in people with schizophrenia. A total of 12 studies identified five primary interventions: Cognitive Behavioral Therapy (CBT), Assertiveness Training (AT), Forgiveness Therapy, Logotherapy, and De-escalation Training. CBT focuses on modifying negative thought patterns that contribute to aggression, while AT helps patients develop assertive communication skills to reduce aggressive responses. Forgiveness Therapy aims to improve psychological well-being by fostering self-acceptance and reconciliation with others. Logotherapy encourages patients to find meaning in their experiences, thereby enhancing emotional regulation. Lastly, De-escalation Training equips psychiatric healthcare providers with non-verbal and verbal techniques to prevent and manage aggressive incidents in clinical settings.

The findings suggest that these interventions may contribute to reductions in violent behavior and associated symptoms. By improving cognitive and emotional regulation, patients can develop healthier coping mechanisms for anger and stress [32]. Furthermore, psychotherapy interventions enhance assertive communication skills, which allow patients to express their emotions and needs without resorting to violence. Beyond safety concerns, these therapies may also lead to improved quality of life, better social interactions, and stronger relationships with family and the broader community [33].

Individual characteristics such as the severity of schizophrenia symptoms, comorbidity with other disorders, and the patient’s motivation to actively participate in therapy can have a significant impact. Interventions tailored to the individual’s clinical needs and symptom severity are likely to be more successful [11]. Furthermore, therapist-related factors such as expertise in the chosen therapeutic technique, experience in treating schizophrenia cases, and the ability to establish a positive therapeutic relationship with the patient also play an important role [32]. In addition, an adequate social environment and family support can increase adherence to therapy and facilitate positive behavioral changes. Consistency in the application of therapeutic techniques, regular monitoring of patient progress, and adaptation of therapy according to individual response also contribute to the effectiveness of the intervention [34].

Among the interventions reviewed, logotherapy appears promising in reducing violent behavior by fostering existential meaning-making. Previous study indicate that this approach helps patients reframe negative experiences and manage emotional distress through existential and spiritual perspectives [35]. Logotherapy encourages patients to seek and find meaning in their life experiences, thereby helping to change the thought patterns and emotional responses that underlie aggressive behavior [26]. Factors that influence the success of logotherapy include adapting therapy to individual needs, the quality of the therapeutic relationship between therapist and patient, and adequate social support. The severity of schizophrenia symptoms and the patient’s level of motivation are also important factors in determining the response to this therapy [36].

CBT has shown efficacy in reducing aggressive behavior by helping patients recognize and modify maladaptive thoughts. The success of CBT is due to its systematic approach in identifying and changing automatic negative thoughts and strengthening support systems in both the family and community [37]. Previous research shows that CBT is effective in reducing symptoms of psychosis and increasing self-control in people with schizophrenia [30]. Factors influencing the success of CBT include active patient involvement, therapist skill and experience, strong social support, and frequency and consistency of therapy sessions [38].

Forgiveness Therapy has demonstrated benefits in enhancing psychological well-being, particularly for family caregivers of individuals with schizophrenia. Studies highlight improvements in self-acceptance and stress reduction, which contribute to better caregiving experiences [3]. The success of this therapy can be explained by a systematic process that helps participants express negative emotions, decide to forgive, and develop new, positive perspectives [28]. Factors influencing the success of this intervention include deep emotional involvement, group support, and a therapy structure that focuses on internal transformation. In addition, the therapist’s skills and sensitivity in facilitating the forgiveness process also play an important role [3]. Other research shows that forgiveness therapy is effective in reducing emotional stress and improving quality of life in various populations [28].

Assertiveness Training has also been associated with reductions in violent behavior by enhancing communication skills and emotional regulation. Research suggests that structured training, including role-playing and therapist demonstrations, facilitates behavioral change [31]. This success can be explained by the systematic and gradual training structure, which includes descriptions of new behaviors, demonstrations by the therapist, group exercises, and role-playing [39]. Factors influencing the success of this intervention include the intensity and duration of training, active involvement of participants, support from the therapy group, and the quality of facilitation by the therapist. Previous studies show that assertiveness training can significantly improve an individual’s ability to manage emotions and reduce aggressive behavior [40]. In addition, family involvement and adequate social support also play an important role in strengthening the positive results of assertiveness training.

De-escalation Training has proven effective in reducing aggression in acute psychiatric settings. Studies report declines in violent incidents following staff training in de-escalation techniques. The success of this intervention relies on comprehensive training content, active participation, institutional support, and continued reinforcement in clinical practice [41]. The intervention uses the comprehensive approach used in training, which includes a theoretical understanding of aggressive behavior, verbal and non-verbal de-escalation techniques, as well as realistic role-playing exercises [42, 43]. Compared with previous research, previous studies also found similar results in reducing aggression through de-escalation training, the latest research adds evidence that this method is consistently effective in a variety of clinical settings [9, 44]. Factors that influence the success of this intervention include the quality of training, active involvement of participants, managerial and operational support in implementing de-escalation techniques, as well as sustainability of training in daily practice [45]. In addition, training tailored to the specific needs of units and patients, as well as ongoing evaluation and feedback, also contribute to the effectiveness of this intervention [46, 47].

Psychotherapy interventions have demonstrated potential in reducing violent behavior in people with schizophrenia, although further large-scale studies are needed to confirm their long-term efficacy and generalizability. Several studies indicate that CBT may contribute to reductions in violent behavior among individuals with schizophrenia; however, variations in study designs and sample characteristics necessitate further investigation to establish definitive conclusions [30, 48]. This is due to CBT’s ability to help patients recognize and change automatic negative thoughts and develop more adaptive coping skills. Other studies have also found similar results in reducing psychosis symptoms and increasing self-control through CBT, these latest findings confirm the consistency and effectiveness of CBT in a variety of clinical settings [49, 50]. In addition, ongoing training for therapists to ensure appropriate application of techniques and responsiveness to individual patient needs is also key to success in reducing violent behavior in people with schizophrenia [51].

The synthesis of findings has been strengthened by incorporating a narrative synthesis comparing various psychotherapy interventions in terms of their mechanisms of action, effectiveness, and relevance to nursing practice. This approach provides a clearer understanding of the comparative value of each intervention, particularly given that a meta-analysis was not feasible due to study variance [52]. CBT operates by identifying and modifying negative thought patterns that contribute to aggression, demonstrating strong evidence for reducing violent behavior in individuals with schizophrenia. Assertiveness Training enhances communication skills and emotional regulation, fostering non-aggressive ways of expressing needs and emotions. Forgiveness Therapy, on the other hand, addresses psychological well-being by promoting self-acceptance and reconciliation, benefiting both patients and caregivers. Logotherapy focuses on existential meaning and acceptance of suffering, which may contribute to reduced aggression through an improved sense of purpose. Meanwhile, De-escalation Training equips healthcare professionals with verbal and non-verbal techniques to manage aggression in psychiatric settings effectively.

Each intervention exhibits varying degrees of effectiveness based on individual characteristics, symptom severity, and therapist expertise [52]. CBT and assertiveness training demonstrate strong efficacy in reducing violent tendencies, while forgiveness therapy and logotherapy contribute significantly to psychological well-being [44]. De-escalation training plays a crucial role in preventing aggressive incidents within healthcare environments, emphasizing its relevance to nursing practice. By comparing these interventions, this review highlights the necessity of tailoring therapeutic approaches to individual patient needs and ensuring adequate training for healthcare professionals to optimize intervention outcomes [27]. Future research should explore multimodal approaches integrating these interventions to enhance overall treatment efficacy and patient quality of life.

Previous studies have shown that psychosocial interventions can reduce the frequency and intensity of violent behavior by helping patients recognize and change negative thoughts, develop adaptive coping skills, and increase psychological well-being and self-acceptance [53, 54]. These findings are in line with previous research which found that CBT and forgiveness therapy were effective in reducing symptoms of psychosis and increasing self-control [45]. Adapting therapy to the specific condition and individual characteristics of the patient also plays an important role in reducing violent behavior in people with schizophrenia [55, 56].

### Limitations

This scoping review has several limitations. The inclusion of studies from only three databases may have restricted the comprehensiveness of the findings, potentially omitting relevant research. Additionally, the focus on recent literature (post-2019) may have led to the exclusion of older but still valuable studies. However, this timeframe was chosen to reflect the most up-to-date advancements in psychotherapy interventions and to ensure the relevance of findings to current clinical practice. While older studies may still offer valuable insights, no significant healthcare system changes prior to 2019 were identified that would substantially impact the applicability of previous findings.

Furthermore, studies published in non-English languages were not included, which may introduce language bias. This decision was made due to resource limitations and the necessity of ensuring a consistent quality of data extraction and interpretation. Future reviews should consider including studies in multiple languages to enhance comprehensiveness. The studies reviewed vary in design, sample characteristics, and outcome measures, which may affect the generalizability of results. While CBT and other interventions show potential in reducing violent behavior, larger-scale randomized controlled trials (RCTs) are needed to validate their long-term efficacy. Additionally, publication bias and differences in measurement tools across studies may influence the reported outcomes. Future research should address these methodological challenges and explore the integration of psychotherapy with pharmacological treatments to optimize patient care.

## Conclusion

The results of this scoping review indicate that 12 studies have explored psychotherapeutic interventions for patients with schizophrenia. The authors identified five types of therapy: logotherapy, assertive therapy, forgiveness therapy, cognitive behavioral therapy (CBT) and social skills training (SST), and assertive communication and de-escalation training. These interventions have demonstrated a significant impact on managing mental health conditions, including reducing violent behavior, improving quality of life, and enhancing patients’ social and emotional functioning. Additionally, psychotherapy interventions contribute to better interpersonal relationships and increased life satisfaction. Several factors influence the effectiveness of psychotherapy in managing violent behavior in individuals with schizophrenia. These include individual factors such as the severity of psychiatric symptoms, motivation for change, and existing cognitive abilities. Furthermore, social support from family and the surrounding environment plays a crucial role in optimizing intervention outcomes.

Despite these findings, caution is warranted when interpreting the results due to the heterogeneity of study designs, variations in sample characteristics, and the limited number of available studies. The generalizability of the findings may be affected by these factors. Future research should focus on addressing these limitations by conducting more rigorous randomized controlled trials (RCTs) with larger sample sizes, long-term follow-up assessments, and comparative studies across different therapeutic approaches to determine the most effective intervention strategies. From a nursing practice perspective, integrating psychotherapy into the care of patients with schizophrenia is essential. Mental health nurses should enhance their competencies in delivering psychotherapeutic interventions such as logotherapy, CBT, and other therapies to improve patient outcomes.

## Data Availability

All data generated or analysed during this study are included in this published article.
